# Synéchies irido-cristallinniennes révélant une spondylarthrite ankylosante

**DOI:** 10.11604/pamj.2014.19.191.4394

**Published:** 2014-10-23

**Authors:** Rajae Derrar, Rajae Daoudi

**Affiliations:** 1 Université Mohammed V, Soussi, Service d'Ophtalmologie A, Hôpital des Spécialités CHU, Rabat, Maroc

**Keywords:** Synéchies irido-cristallinniennes, lombalgies, talalgies, iridocorneal adhesions, back pain, heel pain

## Image en medicine

Patient âgé de 28 ans consultant pour la première fois suite à une baisse d'acuité visuelle ayant comme antécédents des épisodes de rougeur et douleur oculaire récidivants avec sur le plan général des lombalgies et des talalgies. À l'examen, on retrouveau au niveau de l’œil droit une acuité visuelle à 9/10. Son acuité corrigée à 10/10, avec à l'examen à la lampe à fente, une cornée claire avec une chambre antérieure optiquement vide, des synéchies iridocristallinniennes à 6 heures au niveau de l’œil droit (A) avec pigments iriens au niveau de la cristalloide antérieure, le reste de l'examen est normal. L'examen de l’œil gauche trouve une acuité visuelle à 8/10,corrigée à 10/10, des synéchies en trèfle (B) avec pigment sur la cristalloide antérieure,le reste de l'examen est normal. On a suspecté une spondylartrhite ankylosante, une vitesse de sédimentation ainsi qu'une CRP ont été demandées,et sont revenues normales,une radiographie du bassin qui visualise un contour estompé de l'articulation sacro-iliaque ainsi qu'une recherche HLA B27 qui est revenue positive. Le patient a été mis sous traitement anti inflammatoire par voie générale,ainsi que sous mydriatiques vu qu'il n’était pas en poussée inflammatoire, son acuité corrigée était de 10/10.

**Figure 1:**
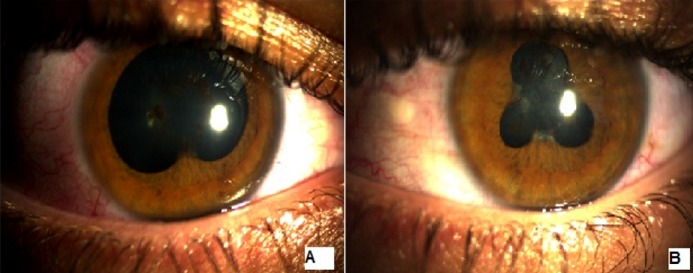
(A) synéchie irido-cristallinienne à 6 heures œil droit, (B) synéchie en trèfle œil gauche

